# Generation, Annotation and Analysis of First Large-Scale Expressed Sequence Tags from Developing Fiber of *Gossypium barbadense* L

**DOI:** 10.1371/journal.pone.0022758

**Published:** 2011-07-28

**Authors:** Daojun Yuan, Lili Tu, Xianlong Zhang

**Affiliations:** National Key Laboratory of Crop Genetic Improvement, Huazhong Agricultural University, Wuhan, Hubei, China; University of Vermont, United States of America

## Abstract

**Background:**

Cotton fiber is the world's leading natural fiber used in the manufacture of textiles. *Gossypium* is also the model plant in the study of polyploidization, evolution, cell elongation, cell wall development, and cellulose biosynthesis. *G. barbadense* L. is an ideal candidate for providing new genetic variations useful to improve fiber quality for its superior properties. However, little is known about fiber development mechanisms of *G. barbadense* and only a few molecular resources are available in GenBank.

**Methodology and Principal Findings:**

In total, 10,979 high-quality expressed sequence tags (ESTs) were generated from a normalized fiber cDNA library of *G. barbadense*. The ESTs were clustered and assembled into 5852 unigenes, consisting of 1492 contigs and 4360 singletons. The blastx result showed 2165 unigenes with significant similarity to known genes and 2687 unigenes with significant similarity to genes of predicted proteins. Functional classification revealed that unigenes were abundant in the functions of binding, catalytic activity, and metabolic pathways of carbohydrate, amino acid, energy, and lipids. The function motif/domain-related cytoskeleton and redox homeostasis were enriched. Among the 5852 unigenes, 282 and 736 unigenes were identified as potential cell wall biosynthesis and transcription factors, respectively. Furthermore, the relationships among cotton species or between cotton and other model plant systems were analyzed. Some putative species-specific unigenes of *G. barbadense* were highlighted.

**Conclusions/Significance:**

The ESTs generated in this study are from the first large-scale EST project for *G. barbadense* and significantly enhance the number of *G. barbadense* ESTs in public databases. This knowledge will contribute to cotton improvements by studying fiber development mechanisms of *G. barbadense*, establishing a breeding program using marker-assisted selection, and discovering candidate genes related to important agronomic traits of cotton through oligonucleotide array. Our work will also provide important resources for comparative genomics, polyploidization, and genome evolution among *Gossypium* species.

## Introduction

Cotton is one of the most important economic crops that provides prevalent natural fiber for the textile industry. The crop is widely cultivated in more than 80 countries, with China, India, the United States of American, and Pakistan being the top four cotton producers (http://www.cotton.org/econ/cropinfo/cropdata/rankings.cfm). Cotton fiber is a single-celled seed trichome, developed from epidermal cells of the ovule. Fiber development consists of four distinct but overlapping stages: fiber cell initiation, expansion/primary cell wall (PCW) synthesis, thickening/secondary cell wall (SCW) synthesis, and maturation/dehydration [1], [2]. Epidermal cells differentiate into fiber cells at approximately the time of anthesis (from −3 to 5 days post-anthesis [dpa]); as a result, only about 30% of the epidermal cells will successfully differentiate into fibers [1], [3]. The initiation stage persists for about 8 days; however, lint fibers usually initiate on the day of anthesis, and fuzz fibers develop at a later stage. During fiber elongation (3–20 dpa), cells elongate rapidly without branching depending on the turgor of the center large vacuole, until the fiber reaches its final length (30–40 mm) [4]. At the same time, PCW begins to synthesize at the surface area of the fiber cell. This is then followed by the synthesis of SCW through the massive deposition of cellulose at 15–40 dpa. The final stage of fiber development, maturation/dehydration (40–60 dpa), is associated with the accumulation of mineral content and decrease in water potential [5]. At maturity, cotton fiber contains about 89% cellulose as is approximately 15 µm in thickness. So, cotton fiber is an excellent model system for studying plant cell elongation, cell wall development, and cellulose biosynthesis [2].

Cotton belongs to the genus *Gossypium* of the family Malvaceae, which consists of 40–45 diploid (2n = 2x = 26) and 5 allotetraploid species (2n = 4x = 52) [6], [7]. The diploid species are grouped into eight genome groups, designed A through G and K. Most modern cotton varieties are allotetraploid species, *Gossypium hirsutum* L. or upland cotton (Gh, AD_1_ genome) and *Gossypium barbadense* L. (Gb, AD_2_ genome). *G. hirsutum*, especially, produces over 90% of the world's fibers because of its higher yield and wider environmental adaptation. However, *G. barbadense* contributes 8% of the world's fibers with its superior properties of silkiness, luster, long staples, and high strength. Thus, *G. barbadense* is a good gene pool for improving the upland cotton fiber quality.

Genomics approaches have been applied to explore the key or predominant expression genes and the mechanism of fiber development in cotton. Several analyses using expressed sequence tags (ESTs) and microarray methods have been performed [8]–[13].

Arpat et al. [9] were the first to take a genomic approach to studying the fiber transcriptome of *G. arboreum* L., a diploid species, at the elongation stage (7–10 dpa). Through in silico expression analysis of 46,603 ESTs, they found that the rapidly elongating fiber cells exhibited significant metabolic activity, cell wall structure, and biogenesis, with the cytoskeleton and energy/carbohydrate metabolism the major functional groups. In a microarray study, they also identified 2553 “expansin-associated” genes down-regulated and 81 “cell wall biogenesis and energy/carbohydrate metabolism-related” genes up-regulated during the developmental switch from PCW to SCW syntheses.

In another excellent and comprehensive work by Udall et al. [10], approximately 185,000 *Gossypium* EST sequences were amassed from 30 cDNA libraries. By sequence comparisons, they found that many cotton exemplars appeared to be involved with transcription, including the high-level categories of transcription factor activity, RNA binding, DNA binding, and nucleotide binding. The most abundant types of Pfam transcriptional domains were MYB DNA-binding, APETALA2, auxin-induced, WRKY DNA-binding, and RING zinc finger domains.

A full-length *G. hirsutum* L. immature ovules (−3 dpa to 3 dpa) cDNA library was constructed and 32,789 high-quality ESTs were generated. By comparing with the existing ESTs and expression analysis, the results determined that transcription factors and phytohormonal regulators were accumulated during early stages of fiber cell development in allotetraploid cotton [11].

By mining the data of cDNA library and generation of ESTs, many actin and tubulin interrelated genes were cloned and shown to have critical roles in the process of fiber development, such as GhTUB1, GhACT1, GhPFN1, GhTUA9, and GhADF1 [8], [14]–[18]. Shi et al. [19] found that 102 metabolic pathways were up-regulated during the fast fiber-elongation period, especially ethylene biosynthesis. Ovule culture *in vitro* indicated that ethylene played a major role in promoting cotton fiber elongation by increasing the expression of sucrose synthase, tubulin, and expansin genes. Recently, many ethylene-responsive species or relevant genes were reported to regulate fiber growth [20]–[23].

A global gene expression profiling study at different stages of fiber development was undertaken on two cotton species for fiber, *G. hirsutum* L. and *G. barbadense* L.. The result showed that secondary metabolism, pectin synthesis, and pectin modification genes were the most statistically significant and differentially expressed categories between the two species and the final fiber property differences between Pima and Upland cotton may largely be determined during early fiber development [24].

Wendel et al. launched important work to study the evolution of spinnable cotton fiber by comparing the expression profile between the cultivar and wild *G. barbadense* [13], *G. longicalyx* and *G. herbaceum* [25], using the microarray method. The result showed that domestication appeared to enhance modulation of cellular redox levels and avoided or delayed the stress-like processes. The cultivar prolonged fiber growth with up-regulation of signal transduction and hormone-signaling genes and down-regulation of cell wall maturation genes. Recent studies also indicated that hydrogen peroxide was important for fiber initiation and elongation [20], [26], [27].

EST large-scale sequencing projects for cotton have been done in several laboratories [9]–[11], [19]. As of December 1, 2010, 375,745 ESTs from *Gossypium* species were deposited in the dbESTs of NCBI GenBank (http://www.ncbi.nlm.nih.gov/dbEST/dbEST_summary.html), including 268,797 ESTs from *G. hirsutum*, 63,577 ESTs from *G. raimondii*, and 41,768 ESTs from *G. arboretum*. Despite the fact that the gene resources of *G. barbadense* were important, few EST resources were deposited for this species compared to *G. hirsutum*, *G. raimondii*, and *G. arboretum*. By the end of December 1, 2010, only 1356 *G. barbadense* ESTs appeared in the NCBI GenBank (dbESTs), including 899 and 333 ESTs, which were submitted by our laboratory in December 2006 and September 2009; the 899 ESTs were generated from the same cDNA library of this research.

We applied genomics approaches to investigate the transcriptional regulation of *G. barbadense* fiber development. A normalized fiber cDNA library (from −2 to 25 dpa) of *G. barbadense* cv. 3–79 (the genetic standard line) was constructed by saturation hybridization with genomic DNA [12]. Random sequencing of clones from the cDNA library generated 10,979 high-quality ESTs, which were assembled into 5852 unique sequences, consisting of 1492 contigs and 4360 singletons. The unique sequences were assigned putative functions based on sequence similarity and Gene Ontology (GO) annotations. The genes governing binding and catalytic activity were more abundant and fiber development was active in the metabolic process, especially in carbohydrate, amino acid, energy, and lipid metabolisms. Moreover, the functions of motif/domain-related cytoskeleton and redox homeostasis were enriched. Furthermore, putative genes involving cell wall biosynthesis and transcription factors were also identified in the fiber development of *G. barbadense*. Finally, the relationships among cotton species with other model plant systems were analyzed. The datasets will benefit the studying of fiber development mechanisms of *G. barbadense* and also be important resources for comparative genomic studies among *Gossypium* species.

## Results

### ESTs sequencing and assembly

Approximately 12,000 clones were successfully single-pass sequenced from the 5′-end. After removal of vector, poly-A, and contaminating microbial sequences as well as those less than 100 bp in length, 10,979 ESTs passed the quality control for high confidence base call (Q20), and were deposited in GenBank under the accession no. GR706801–GR716890, EE592400–EE593286, EH122780, and EH122781. The average length of all 10,979 ESTs was 643 bp; the longest was 1184 bp. The ESTs were clustered and assembled into 5852 unique sequences (putative unigenes), consisting of 1492 (25.5%) contigs and 4360 (74.5%) singletons, and the EST redundancy of this library was 53.3%. The average length was 706 bp for unique sequences, 915 bp for contigs, and 633 bp for singletons (Table 1). The lengths of 488 (32.7%) contigs were longer than 1000 bp and the largest percentage of unigenes were 800–899 bp (1327, 22.7%). The detailed length distributions of ESTs, unigenes, contigs, and singletons were shown in Table S1 and Figure S1. The mean G/C content of unigenes was 43.1%, which was approximately equivalent to *Arabidopsis* (43.2%) and much lower than rice (51.4%) [28], [29].

**Table 1:** EST sequence and assembly statistics.

Total number of sequence reads	11,180
High-quality sequences (Q>20 and at least 100 bp in length)	10,667
After removal of vector, poly-A, contaminating microbial sequences, and very short sequences (>100 bp) (GI:GR706801–GR716890)	10,090
The sequence were submitted to NCBI at 2006 (GI:EE592400–EE593286, EH122780, EH122781)	889
Average EST size after trimming (bp)	643
Longest sequence after trimming (bp)	1184
Total number of assembled sequences	10,979
Number of contigs	1492
Average number of ESTs in contigs	4.4
Number of singletons	4360
Number of unique sequences	5852
Average length of unique sequences (bp)	706
Average length of contigs (bp)	915
Average length of singletons (bp)	633
Longest length of unique sequences (bp)	3214

**Figure 1:**
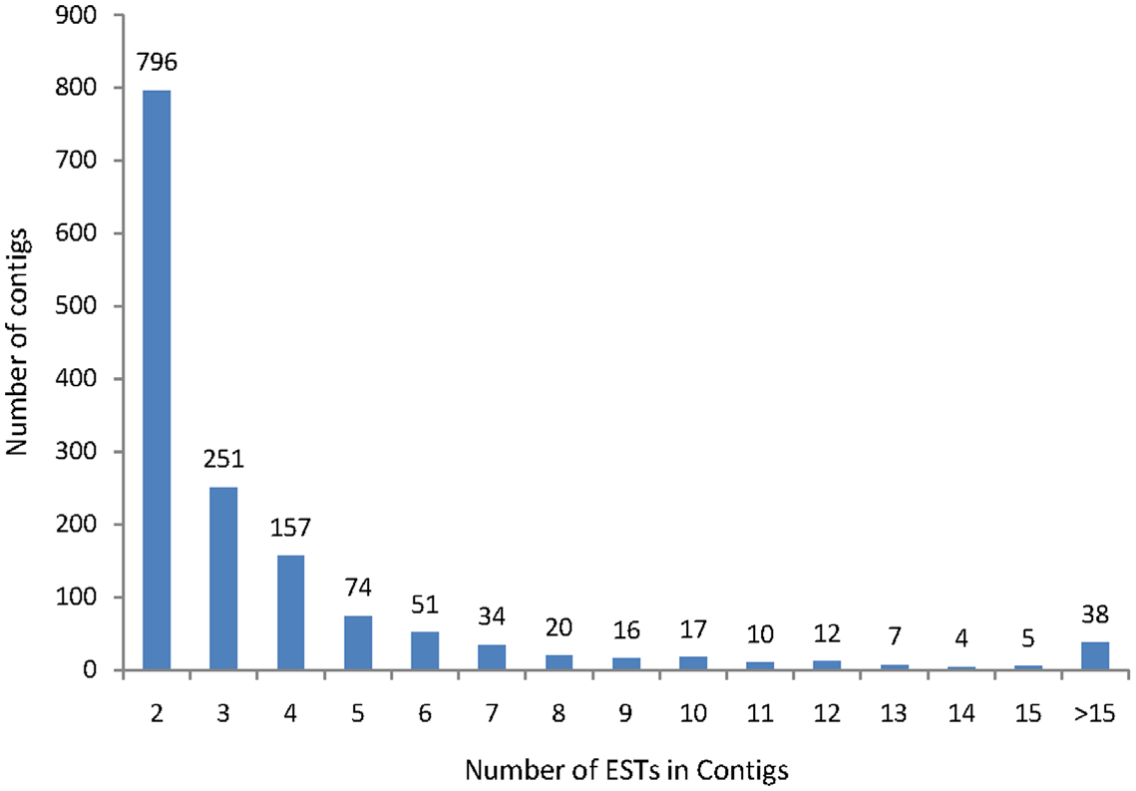
Distribution of 1492 contigs based on the number of clustered ESTs.

The average number of ESTs per contigs was 4.4 with the maximum being 410. Of the 1492 contigs, 445 (29.8%) contigs have four or more transcripts (Fig. 1), suggesting that the redundancy rate was relatively low in this normalized library. Table 2 and Table S2 (each of these clusters contained ≥10 EST copies and represented 21.7% of the total number of ESTs obtained) indicated the most abundantly expressed genes, encoding key fiber proteins such as arabinogalactan protein (AGP) and fasciclin-like arabinogalactan protein (FLA) (CO000009, CO000056,CO000570, CO000039, CO000446, CO000130, including 227 ESTs), tubulin (CO000092, CO000013, CO000075, CO000195, CO000081, including 236 ESTs), translation elongation factor 1A (EF1A) (CO000108, CO000125, CO000270, CO000677, including 62 ESTs), fiber protein (CO000128, CO000259, CO000190, including 86 ESTs), or important enzymes for redox, such as dehydrogenase (CO000230, CO000043), reductase (CO000249, CO000612), and ascorbate peroxidase (CO000002). There were many novel sequences without function annotation, such as CO000118, CO000042, CO000165, and CO000131 (Table S2).

**Table 2:** Twenty highly abundant genes in the 10,979 ESTs.

Contig no.	Contig length	No. ESTs	Putative function
CO000020	3214	410	Putative senescence-associated protein
CO000092	1882	91	α-tubulin
CO000193	2009	91	E6-3 protein kinase
CO000009	1202	88	Fasciclin-like arabinogalactan protein
CO000013	1807	66	α-tubulin
CO000006	669	65	High-glycine tyrosine keratin-like protein
CO000128	643	63	Fiber protein Fb28
CO000056	1047	56	Fasciclin-like arabinogalactan protein
CO000170	936	54	Lipid-binding protein
CO000145	1937	52	α-expansin
CO000026	1207	43	Protodermal factor
CO000218	923	43	Fblate-2 gene
CO000075	1819	42	α-tubulin 6
CO000083	823	36	Glycine-rich RNA-binding protein
CO000298	1873	33	18S ribosomal RNA gene
CO000364	1257	32	Fblate-2
CO000017	1296	29	Chitinase-like protein
CO000570	1066	29	Fasciclin-like arabinogalactan protein 3
CO000095	1300	28	Dehydration-induced protein RD22-like protein
CO000185	1126	27	Ubiquitin

To determine if the putatively enriched genes were highly expressed truly, we performed reverse transcription polymerase chain reaction (RT-PCR) (File S1 and Table S3). Results shown in Figure S2 confirmed the high level expression for the genes represented by the ten contigs, which suggested they were all truly abundant and expressed in a tissue or a developmental stage at least in *G. barbadense* or *G. hirsutum*.

### Functional annotation and classification

The 5852 unique sequences were used in a blastx search against the non-redundant protein sequence (nr) database in GenBank. A total of 4862 (83.1%) unigenes had significant hits (E-value≤10^−5^) (Table S4). However, of the 4862 significant hits unique sequences, only 2165 (37.0%) showed similarities to proteins of known function, 2697 (46.1%) showed similarities to predicted proteins of unknown function, and 990 (16.9%) showed no significant similarity to any sequences contained in the nr database (Table S5). The organism distribution of the unigenes best blastx hits was 1240 (25.5%) of *Ricinus communis*, 1106 (22.7%) of *Vitis vinifera*, 1090 (22.4%) of *Populus trichocarpa*, 136 (2.8%) of *Arabidopsis thaliana*, 51 (1.1%) of *Medicago truncatula*, 38 (0.8%) of *Oryza sativa*, and 378 (7.7%) of *Gossypium* (including 305 *G. hirsutum*, 41 *G. barbadense*, 21 *G. arboreum*, and 8 *G. raimondii*) (Table S6). However, of the 2697 predicted proteins of unknown function (Table S7), 1094 (40.6%) were from *V. vinifera*, 1014 (37.6%) were from *P. trichocarpa*, and 336 (12.5%) were from *R. communis* (Table S8). Then, 990 sequences with no blastx hits were searched for similarities at the nucleotide level (non-redundant nucleotide sequence nt database in GenBank); only 205 sequences shared homology with the genes registered in the NCBI nt database (Table S9), including 106 sequences similar to cotton (Table S10). However, 785 unigenes (13.4%) remained unidentified, which could be considered as novel or specific genes in *G. barbadense*. This result might have occurred because some sequences were too short (the average length of 785 sequences was only 367 bp and 506 sequences are shorter than 400 bp) (Table S11). In order to get more known information, unique sequences were also used in a blastx search against swissprot database. A total of 67.2% (1813 sequences) of 2697 sequences which showed similarities to predicted proteins of unknown function in nr database, were similar to known protein sequences in swissprot database (Table S7). However, 990 sequences without similarity to any sequences in the nr database were also not found any similarity sequences in swissprot database.

#### Gene ontology annotation

Gene Ontology (GO) annotation was performed with BLAST2GO [30], [31] based on comprehensive information with sequence similarity against NCBI non-redundant (nr) protein database, InterProScan result and plant-related GO terms slimmed. A total of 4461 (76.2%) unigenes were functionally classified in one or more ontologies, and 3492 (59.7%) of the 5852 unigenes with assigned GO terms had molecular functions, 3137 (53.6%) were involved in a biological process, and 3012 (51.5%) were cellular components; 1830 (31.3%) unique sequences were classified in three ontologies. Among the 1391 unigenes without assigned GO terms, 990 did not have sequences with blastx results, 167 without annotation results, and 234 without GO terms.

**Figure 2:**
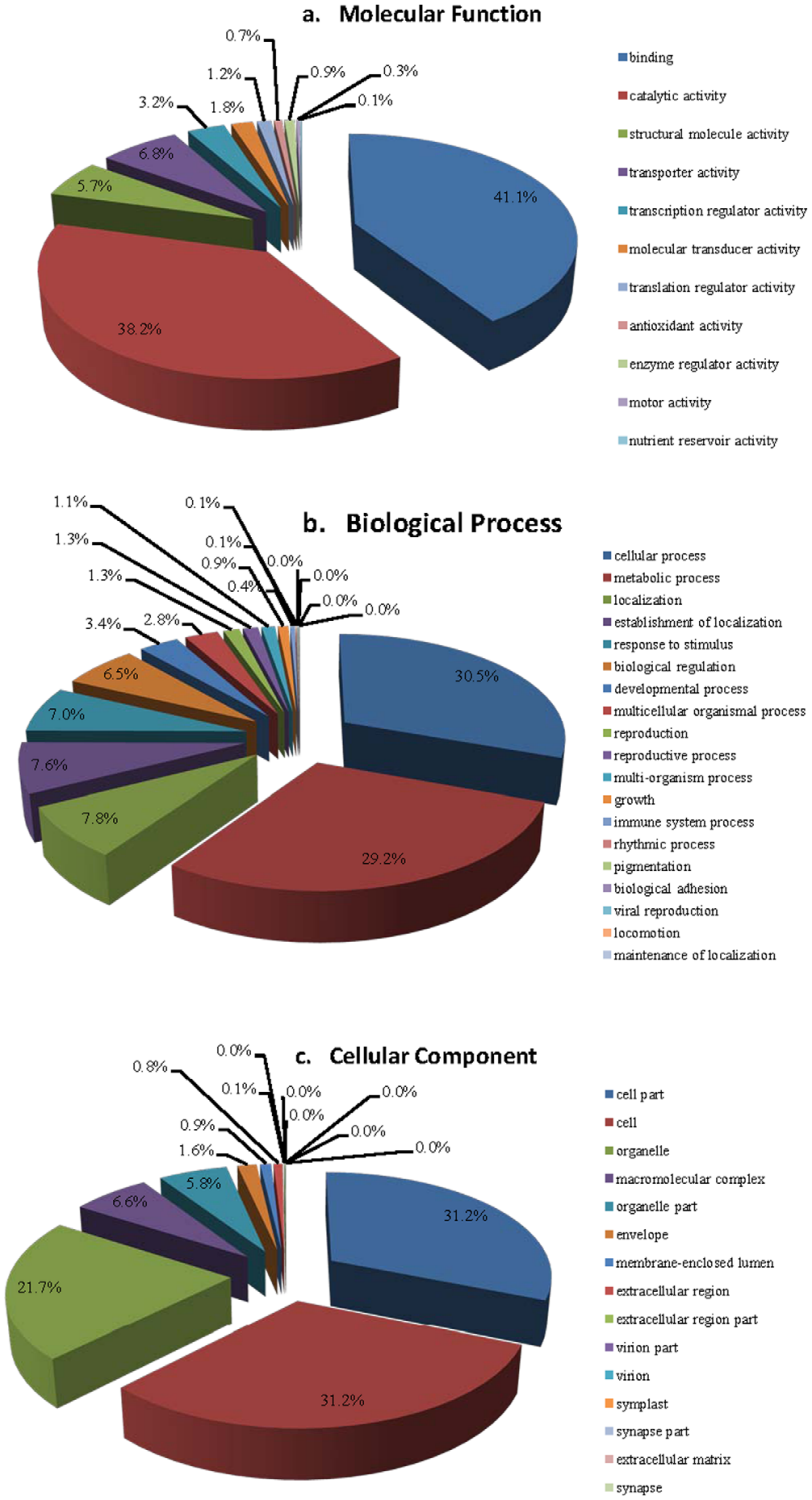
Functional classifications for the 5852 unigenes that were assigned with GO terms (second level GO terms). The three GO categories, biological process (a), molecular function (b), and cellular component (c) are presented.

The three categories of GO terms fell predominantly into two or three subcategories (Fig. 2). In the molecular function (MF) class (second level GO terms, Fig. 2a) the majority of the GO terms were grouped into two categories, namely, binding (GO:0005488, 41.1%) and catalytic activity (GO:0003824, 38.2%). In binding, nucleotide binding (GO:0000166, 10.8%), protein binding (GO:0005515, 10.7%), ion binding (GO:0043167, 8.8%), and nucleic acid binding (GO:0003676, 8.6%) were the mostly enriched terms at third level (Figure S3a). Yet, the highly enriched GO terms in catalytic activity included hydrolase activity (GO:0016787, 11.6%), transferase activity (GO:0016740, 10.6%), and oxidoreductase activity (GO:0016491, 7.3%) (Figure S3a).

Considering the biological process (BP) class (second level GO terms, Fig. 2b), the vast majority were involved in the cellular process (GO:0009987, 30.5%) and metabolic process (GO:0008152, 29.2%). The cellular metabolic process (GO:0044237), primary metabolic process (GO:0044238), and macromolecule metabolic process (GO:0043170) were included in metabolic processes (third level GO terms) and represented the 15.6%, 14.9%, and 10.9% of the GO terms on that level, respectively (Figure S3b).

Furthermore, for the cellular component (CC) class (second level GO terms, Fig. 2c) the assignments were mostly given to cell part (GO:0044464, 31.2%), cell (GO:0005623, 31.2%), and organelle (GO:0043226, 21.7%) (Fig. 2c).

#### Annotation augmentation using InterProScan

The function motif/domain for ESTs and unigenes were obtained through InterProScan [32] use of BLAT2GO bioinformatics tool [30], [31]. The most common InterPro families are presented in Table 3. Result showed that there were 1896 unigenes with 1379 InterPro families recognized. Of these 1896 sequences, 41 had no similarity with any sequence in the NCBI nr database. The most frequent family is NAD(P)-binding (IPR016040) with 39 unigenes, followed by the EGF-like region (IPR013032, 37 unigenes), von Willebrand factor (IPR001007, 34 unigenes), Ras GTPase (IPR001806, 34 unigenes), and small GTP-binding protein (IPR005225, 32 unigenes). The families related to tubulin (including 6 members and 98 unigenes), ubiquitin (including 11 members and 88 unigenes), thioredoxin (including 7 members and 86 unigenes), and ferredoxin (including 9 members and 63 unigenes) are also abundant (Table S12).

**Table 3:** The most frequent InterPro families found in *G. barbadense* EST library.

InterPro no.*	Description	Total of unigenes	Total of ESTs
IPR016040	NAD(P)-binding	39	54
IPR013032	EGF-like region, conserved site	37	52
IPR001007	von Willebrand factor, type C	34	42
IPR001806	Ras GTPase	34	26
IPR005225	Small GTP-binding protein	32	59
IPR000719	Protein kinase, core	31	27
IPR006058	2Fe-2S ferredoxin, iron sulfur-binding site	31	38
IPR011009	Protein kinase-like	29	32
IPR012335	Thioredoxin fold	29	48
IPR012336	Thioredoxin-like fold	27	45
IPR000217	Tubulin	26	114
IPR009072	Histone-fold	26	39
IPR000608	Ubiquitin-conjugating enzyme, E2	25	49
IPR016135	Ubiquitin-conjugating enzyme/RWD-like	25	49
IPR000020	Anaphylatoxin/fibulin	24	29
IPR012677	Nucleotide-binding, α-β plait	24	42
IPR017442	Serine/threonine protein kinase-related	24	26
IPR013753	Ras	23	36
IPR000626	Ubiquitin	22	30
IPR007125	Histone core	22	34
IPR000504	RNA recognition motif, RNP-1	21	37

*The list included the families with >21 UniGenes.

#### KEGG pathway assignment

In addition, we also annotated unique sequences using KAAS (KEGG Automatic Annotation Server) [33]. Results showed a enrichment for the category metabolism (1303, 22.3%), followed by the categories genetic information processing (GIP, 516, 8.8%), cellular process (CP, 342, 5.8%), environmental information processing (EIP, 106, 1.8%), and organismal systems (OS, 314, 5.4%). As summarized in Table 4, carbohydrate (28.3% of metabolism), amino acid (18.0% of metabolism), energy (14.0% of metabolism), and lipid (10.0% of metabolism) metabolisms were major contributors among the subsets of metabolism. In the category of GIP, folding, sorting, and degradation (44.4% of GIP) and translation (40.7% of GIP) were the majority as opposed to transcription (9.9% of GIP). In the category of EIP, the vast majority were involved in signal transduction (95.3% of EIP). Transport and catabolism (44.7%), cell growth and death (31.3%), and cell communication (18.4%) constitute the majority of CP category. Other than the above categories, the major constitution of organismal systems were endocrine system (25.5%), immune system (21.0%), and environmental adaptation (13.7%).

**Table 4:** The distribution of the KEGG pathway.

	Pathway	Total of unigenes	Percent of unigenes (%)	Percent of categories (%)
**Metabolism(1303,22.27%)**	Carbohydrate metabolism	369	6.3	28.3
	Energy metabolism	183	3.1	14.0
	Lipid metabolism	131	2.2	10.1
	Nucleotide metabolism	49	0.8	3.8
	Amino acid metabolism	234	4.0	18.0
	Metabolism of other amino acids	67	1.1	5.1
	Glycan biosynthesis and metabolism	16	0.3	1.2
	Metabolism of cofactors and vitamins	60	1.0	4.6
	Metabolism of terpenoids and polyketides	38	0.7	2.9
	Biosynthesis of other secondary metabolites	69	1.2	5.3
	Xenobiotics biodegradation and metabolism	87	1.5	6.7
**GIP(516,8.82%)**	Transcription	51	0.9	9.9
	Translation	210	3.6	40.7
	Folding, sorting, and degradation	229	3.9	44.4
	Replication and repair	26	0.4	5.0
**EIP(106,1.81%)**	Membrane transport	3	0.1	2.8
	Signal transduction	101	1.7	95.3
	Signaling molecules and interaction	2	0.0	1.9
**CP(342,5.84%)**	Transport and catabolism	153	2.6	44.7
	Cell motility	19	0.3	5.6
	Cell growth and death	107	1.8	31.3
	Cell communication	63	1.1	18.4
**OS(314,5.37%)**	Immune system	66	1.1	21.0
	Endocrine system	80	1.4	25.5
	Circulatory system	24	0.4	7.6
	Digestive system	18	0.3	5.7
	Excretory system	21	0.4	6.7
	Nervous system	32	0.6	10.2
	Sensory system	18	0.3	5.7
	Development	12	0.2	3.8
	Environmental adaptation	43	0.7	13.7

GIP: Genetic Information Processing; EIP: Environmental Information Processing; CP: cellular process; OS: organism systems.

### Comparisons to the other cotton species and model plant species

**Figure 3:**
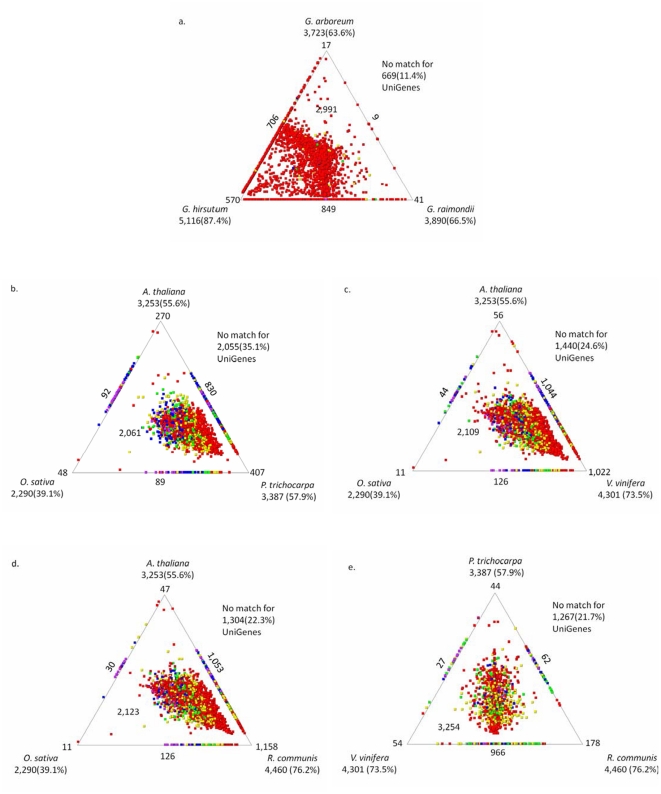
SimiTri profile of UniGenes. The 5852 unigenes were searched against the nucleotide databases for ESTs (a) or protein (b, c, d, e) using blastn (a) or blastx (b, c, d, e) (E-value≤10^−5^). The color was coded based on the highest BLAST score as: red >300; yellow >200; green >150; blue >100, and purple <100.

As of December 2010, 375,745 ESTs from all *Gossypium* species had been deposited in GenBank. To identify the *G. barbadense* species-specific sequence relative to other cotton species, the unigenes were used as queries in a blastn search against three databases including 268,797 ESTs of *G. hirsutum*, 63,577 ESTs of *G. raimondii*, and 41,768 ESTs of *G. arboretum* downloaded from GenBank, respectively. The two-dimensional display of relative similarity relationships between *G. barbadense* with three other cotton species was showed by the program SimiTri [34] (Fig. 3a). A total of 5183 (88.6%) unigenes had similarity with one or more species, with 2991 (51.1%) unigenes shared by four cotton species. However, 570, 41, and 17 unigenes shared only one species by *G. hirsutum* (AD-genome), *G. raimondii* (D-genome), *G. arboretum* (A-genome), respectively. A total of 3723 (63.6%) of the cluster sequences had homologues in *G. arboreum*, 5116 (87.4%) in *G. hirsutum*, and 3890 (66.5%) in *G. raimondii*, and 669 (11.4%) had no significant match to any sequence in the current EST databases of cotton. In order to validate the *G. barbadense* specific unigenes, five of them were selected from Table S13 to analyze their expression patterns by RT-PCR method. The details and results are shown in File S1, Table S3 and Figure S2. The results showed they were specifically or predominantly expressed in the tissues of *G. barbadense*, especially the sequences CO001089, 02-D20 and 44-O06, which were very lowly or hardly expressed at the fiber of *G. hirsutum* (Figure S2).

In addition, the unigenes were compared with the protein sequences of *Arabidopsis*, *Oryza*, *Populus*, *Vitis*, and *Ricinus* using blastx. The results (Fig. 3b–e) found, overall, 39.1%, 55.6%, 57.9%, 73.5%, and 76.2% of unigenes had similarity with *Oryza*, *Arabidopsis*, *Populus*, *Vitis*, and *Ricinus*, respectively.

### Cell wall-related protein families

*Gossypium* is a cell wall model species, so the unigenes were assessed by blastx against the Cell Wall Navigator (CWN) database [35] to identify cell wall-related genes. In the 5852 unigenes, 282 (4.8% of unigenes) sequences had homologs (blastx, E-value≤10^−5^) in the CWN, including 915 ESTs (8.3% of ESTs) classified into 19 cell wall metabolism categories (Table 5 and Table S14). The most abundant cell wall gene category was arabinogalactan protein (AGP) genes, with a total of 237 ESTs (4.0%). Other cell wall-related genes in the most highly abundant genes include NAD-dependent epimerase/dehydratase superfamily (NSI), expansins (EXP), glycosyl transferases (pectin synthesize) (GT8), yieldins (GH18), glycoside hydrolases 9 (GH9), pectin methyl esterases (PME), and leucine-rich repeat extensins (LRX).

**Table 5:** The categories of cell wall-related genes.

Categories	Total of ESTs	Total of unigenes	Redundancy
1.1 Sugar 1-kinases (S1K)	1	1	1.0
1.2 Nucleotide-sugar pyrophosphorylases	16	10	1.6
1.3 Nucleotide-sugar interconversion enzymes	186	36	5.2
2.1 Cellulose and galactomannan	31	11	2.8
2.2 Hemicellulose	33	19	1.7
2.3 Callose	5	4	1.3
2.4 Other glycosyl transferases	64	49	1.3
3.1 Cell expansion	128	31	4.1
3.2 Hemicellulose reassembly	24	9	2.7
3.3 Glycoside hydrolases	79	37	2.1
3.4 Lyases	29	8	3.6
3.5 Esterases	33	20	1.7
4.1 Hydroxyproline-rich glycoproteins (HRGP)	8	6	1.3
4.2 Leucine-rich repeat extensins (LRX)	30	21	1.4
4.3 Proline-rich proteins (PRP)	3	2	1.5
4.4 Glycine-rich proteins (GRP)	0	0	0.0
4.5 Arabinogalactan proteins (AGP)	237	12	19.8
5.1 Glycoprotein fucosyltransferases (GFT)	1	1	1.0
5.2 Glycosyl transferases 21A (GT31a)	3	3	1.0
5.3 Glycosyl transferases 31B (GT31b)	4	2	2.0
Total	915	282	3.2

Some cell wall genes were highly abundant (≥10 ESTs), including NAD-dependent epimerase/dehydratase family protein, arabinogalactan protein, fasciclin-like arabinogalactan-protein (FLA9 and FLA11), expansins, endochitinase 2 precursor, membrane-anchored endo-1,4-β-glucanase, DTDP-glucose 4,6-dehydratase, pectate lyase, UDP-glucuronate decarboxylase 2, and cellulose synthase. Other cell wall-related genes including pectin methylesterases (PMEs), FLA6, and xyloglucan endotransglycosylases (XETs) were moderately abundant (5∼9 ESTs).

### Identification of putative transcription factors

PlantTFDB 2.0, a comprehensive plant transcription factor (TF) database for 49 species, including 53 319 putative TFs and 58 families [36], was used to identify putative TFs. Blastx searches revealed 736 (12.6% of unigenes, including 1317 ESTs) with matches in PlantTFDB at E-value≤10^−5^. The organism distribution of the best unigenes blastx hits was 15.8% of *G. hirsutum*, 10.9% of *V. vinifera*, 7.3% of *Brachypodium distachyon*, 7.1% of *O. sativa subsp. Indica*, 5.3% of *Zea mays*.

The 736 TFs fell into 53 families. The most abundant TF family was the bZIP group (71, 9.6%) followed by the MYB-related (65, 8.8%), bHLH (50, 6.8%), C2H2 (46, 6.3%), MYB (44, 6.0%), C3H (43, 5.8%), NAC (32, 4.3%) and WRKY (31, 4.2%) families (Table 6). The distribution of TF families in *G. barbadense* and seven related species were listed in Table S15. Compared with other model species, in the high-frequency TF families, bZIP (71, 9.6%), MYB-related (65, 8.8%), C2H2 (46, 6.3%), C3H (43, 5.8%), s1fa-like (28, 3.8%), and Dof (19, 2.6%) families showed relatively higher frequency in *G. barbadense*, whereas bHLH (50, 6.8%), ERF (22, 3.0%), LBD (4, 0.5%), and HSF (3, 0.4%) families were lower. However, HB-PHD, LFY, RAV, SAP, and STAT families were not detected in our datasets (Table S15).

**Table 6:** The most abundant putative transcriptional factors(TFs).

TF family	TF description	Total of ESTs	Total of unigenes	Redundancy^a^	Percent (%)^b^
bZIP	Basic leucine zipper (bZIP) motif	71	71	1.0	9.6
MYB related	N-terminal myb-domain	370	65	5.7	8.8
bHLH	basic/helix-loop-helix domain	50	50	1.0	6.8
C2H2	Zinc finger, C2H2 type	46	46	1.0	6.3
MYB	Myb-like DNA-binding domain	44	44	1.0	6.0
C3H	Zinc finger, C-x8-C-x5-C-x3-H type	43	43	1.0	5.8
NAC	No apical meristem (NAM) protein	124	32	3.9	4.3
WRKY	WRKY DNA-binding domain	31	31	1.0	4.2
S1Fa-like	negative cis-element S1F binding site	68	28	2.4	3.8
G2-like	Golden 2-like (GLK)	25	25	1.0	3.4
ERF	single AP2/ERF domain	22	22	1.0	3.0
Trihelix	Trihelix DNA-binding domain	50	22	2.3	3.0
Dof	DNA binding with one zinc finger	19	19	1.0	2.6
HD-ZIP	HD domain with a leucine zipper motif	17	17	1.0	2.3
ARF	Auxin response factor	16	16	1.0	2.2
M-type	MADS-box transcription factors	16	16	1.0	2.2
FAR1	Far-Red-impaired Response 1	15	15	1.0	2.0
HB-other	Homeobox domain	14	14	1.0	1.9
GRAS	three initially identified members, GAI, RGA and SCR	12	12	1.0	1.6
MIKC	MIKC-type MADS-box gene include three more domains intervening (I) domain, keratin-like coiled-coil (K) domain, and Cterminal (C) domain	12	12	1.0	1.6
NF-YC	Nuclear Factor Y subunits C proteins	41	12	3.4	1.6
ARR-B	Arabidopsis response regulators(ARRs) with a Myb-like DNA binding domain(ARRM)	10	10	1.0	1.4
NF-X1	NF-X1 type zinc finger	40	10	4.0	1.4

a Redundancy is (Total of ESTs)/(Total of Unigenes).

b Percent is (Total of unigenes)/(Total of putative TFs, 736).

### Analysis of microsatellite repeats

Microsatellites or simple sequence repeats (SSRs) are one of the best genetic markers for mapping purposes [37] and most useful for molecular marker systems in plant breeding [38], especial EST-SSR with high efficiency and low cost [39]. In this study, a total of 497 SSRs were identified in 460 (7.9%) unigenes at a frequency of 1 in 8.3 kb. These SSRs have been used to construct a genome-wide SSR genetic map [40]. A total of 26, 4, and 1 sequences were examined in 2, 3, and 4 SSR loci, respectively. The SSRs found are summarized in Table 7 and Table S16. Among all the repeat types, trinucleotide repeats were the most abundant SSR types (187, 37.6% of EST-SSR), followed by pentameric SSRs (98, 19.7%), dimeric SSRs (94, 18.9%), hexameric SSRs (81, 16.3%), and tetrameric SSRs (37, 7.4%). The most frequent dinucleotide motif was AT/TA (44, 46.8%) and the second was AG/CT (36, 38.3%). For trinucleotide, AAG/CTT (55, 29.4%), ATC/GAT (34, 18.2%), and ACC/GGT (27, 14.4%) were the three most frequent motifs (Table S16).

**Table 7:** Features of SSRs.

Total number of sequences examined	5852
Total number of identified SSRs	497
Number of SSR-containing sequences	460
Number of sequences containing more than one SSR	31
Total size of examined sequences (kb)	4125.7
Average distance (kb)	8.3
**Distribution of SSRs**	
Number of dinucleotide repeats	94 (18.9%)
Number of trinucleotide repeats	187 (37.6%)
Number of tetranucleotide repeats	37 (7.4%)
Number of pentanucleotide repeats	98 (19.7%)
Number of hexanucleotide repeats	81 (16.3%)

## Discussion

### The enlargement of the *G. barbadense* EST database is a good supplement for fiber development work in cotton

Although there are two cultivated tetraploid species, *G. hirsutum* and *G. barbadense*, few researchers investigated *G. barbadense* because it is less cultivated. EST sequencing is an efficient and relatively low-cost approach for gene discovery and annotation, detection of gene expression, genome and physical mapping, and molecular marker development [41], especially important for the organisms whose whole genome sequencing is currently uncompleted. In *Gossypium*, the highest number of ESTs in the NCBI GenBank was from *G. hirsutum*. Although *G. barbadense* has superior properties, its genomic resources are relatively undiscovered. Only 124 ESTs appeared in the NCBI GenBank (dbESTs), excluding 899 and 333 ESTs that were submitted by our laboratory in December 2006 and September 2009. In this study, we produced more than 10,000 high-quality ESTs from a normalized fiber cDNA library (from −2 to 25 dpa) of *G. barbadense* cv. 3–79 (the genetic standard line). Most of the ESTs were from fiber initiation and elongation developmental stages, and only a few were involved with the SCW synthesis period (http://www.ncbi.nlm.nih.gov/UniGene/lbrowse2.cgi?TAXID=3635&log=breadcrumbs). The ESTs were assembled into 5852 unigenes and annotated through a similarity search. Annotation results showed that many previously reported cotton fiber active or key genes were included in these libraries, such as AGPs and FLAs [42]–[44], GhTUB, GhTUA [15], [17], actin-related genes (including actin-depolymerizing factor and profilin ) [14], [16], [18], [45]–[47], and GhEF1As [48]. A normalized cDNA library was an efficient tool for gene identification because it reduced the frequencies of prevalent mRNAs while enriching the rare ones. E6 and aquaporin PIP2-2, the most redundant transcriptions in cotton fiber [9], were only 13 and 19 clones in our ESTs, respectively. So, the first large-scale and publicly available ESTs from *G. barbadense* will be an important genomic resource to identify novel genes, especially for low-redundant ones through all the fiber development stages.

### *G. barbadense* has a specific expression profile compared with other plant model systems

In our work, 5067 (86.6%) unigenes had significant blastx or blastn hits after BLAST annotation although 785 unigenes (13.4%) remained unidentified and could be considered as novel or specific genes in *G. barbadense*. The most organism distribution of the unigenes best blastx hits is in *R. communis* (25.5%), followed by *V. vinifera* (22.7%), *P. trichocarpa* (22.4%), and *Gossypium* (7.7%) (Table S6). *A. thaliana* and *O. sativa* were the best model systems for plant biology; however, the species at the top the blastx result were only 2.8% and 0.8% from *A. thaliana* and *O. sativa* in these datasets (Table S6). In addition, the unigenes were also compared with the protein sequences of *A. thaliana*, *O. sativa*, *P. trichocarpa*, *V. vinifera*, and *R. communis* using blastx. As shown using SimiTri [34] software in Fig. 3 (b, c, d, e), 39.1%, 55.6%, 57.9%, 73.5%, and 76.2% of unigenes have similarity with *O. sativa*, *A. thaliana*, *P. trichocarpa*, *V. vinifera* and *R. communis*, respectively. When compared with *A. thaliana*, *O. sativa*, and *P. trichocarpa* (Fig. 3b), the closest was *P. trichocarpa* and the most distant was *O. sativa*. Moreover, the mean G/C content of unigenes was approximately equivalent to *A. thaliana* and much lower than rice. Through sequencing bacterial artificial chromosomes (BACs) and analyzing the phylogenetic tree, Yu [49] also found that cotton was a nearer relative to poplar than the others. *O. sativa* is a monocot and *A. thaliana*, *P. trichocarpa*, and cotton are dicots, which may account for the differences in similarity between them in sequence. Although both *G. barbadense* and *A. thaliana* are Eurosid II clade (*P. trichocarpa* is Eurosid I) (http://www.mobot.org/MOBOT/research/APweb/), some *G. barbadense* genes appeared to be more similar to *P. trichocarpa* than *A. thaliana* as shown in Fig. 3b. *P. trichocarpa* is a perennial tree and the bulk of the biomass of trees is cellulose [50]. In addition, the ESTs are generated from cotton fiber tissues, which contain about 89% cellulose at maturity. Furthermore, through the analysis of genome sequence of woodland strawberry, Vladimir et al. proposed that poplar has to place into Malvidae clade and not Fabidae [51]. Compared with *Ricinus*, which is the same order with poplar (Malpighiales), cotton has more similar genes to *R. communis* (76.2%) than *P. trichocarpa* (57.9%) (Fig. 3d). The reason may be that fiber is developed from seed trichome and cotton seed also has high-quality oil materials, and *R. communis* is also an important oil crop. To our surprise, cotton also has high similarity with *V. vinifera* (73.5%) (Fig. 3c), which is the most distant relative in the plant phylogenetic tree. Although compared with *P. trichocarpa*, *V. vinifera*, and *R. communis* (Fig. 3e), the total number of similar genes between cotton with the three species is different, respectively; however, 3254 of common sequences were the same distance to three organisms. Based on the above results, it is difficult to decide on a best model plant to study cotton or cotton fibers, so cotton genome sequencing is vital and urgent.

### Transcription factors (TFs), expansins, cell cytoskeletons, and reactive oxygen species (ROS)-related genes are highly enriched during fiber development

Despite the fact that cotton fiber is single-celled trichome, the development of fiber is an exceptionally genetic complex and more dynamic gene network [27], [52], [53]. The development of fiber was enriched by transcription factors and phytohormonal regulators [11], [19], [54], [55]. Yang et al. found that the frequency of putative TFs was approximately 10% in the *G. hirsutum* fiber initiation library (GH_TMO), which was significantly higher than that in CGI6 (Cotton Gene Index version 6, approximately 4.7%), CGI7 (approximately 5.0%), and the *Arabidopsis* proteome (approximately 6.3%) [11]. However, the frequency of putative TFs in this cDNA library is about 12.5%, which is much higher than that in GH_TMO. The result showed that the development through stages in *G. barbadense* may be enriched in TFs especially. TFs play critical roles in the regulation of cellular pathways in the response to biotic and abiotic stimuli and intrinsic developmental processes. The enrichment of TFs perhaps enhanced modulation of cellular redox levels and the avoidance or delay of stress-like processes, which prolonged the elongation period of *G. barbadense* characterized as longer fibers [13]. The difference of high-frequency TFs might be caused by using the different databases and organisms. TFs of 49 plant species were used in this study, however, only *Arabidopsis* TFs were used in the study by Yang et al. [11]. Only 55.6% of the ESTs in *G. barbadense* were similar to *Arabidopsis*, which may be not the best model system for cotton.

Rapid elongation of fiber cells is associated with cell turgor pressure [4]. Besides maintaining the high cell turgor, plasmodesmatal regulation and cell wall reassembly are also important for fiber elongation [56], [57]. Plant expansins are a group of extracellular proteins that directly modify the mechanical properties of cell walls, enable turgor-driven cell extension, and likely affect length and quality of cotton fibers [58], [59]. The expansin-related genes were at a very high level in our library. As shown in Table S2, two contigs including 67 ESTs are expansin genes. In Table S14, of 128 EST-related expansins, 53 ESTs (21 unigenes) are yieldins (glycosyl hydrolase family 18 protein, GH18). Yieldins lower the yield threshold of the minimum of tensile force needed to extend the cell wall [60] and were expressed in hypocotyl tissue prior to elongation activated at low pH [61]. Yieldins were first discovered or highlighted in fiber development and may be a key family protein for fiber elongation. Cell cytoskeletons also play important roles in plant cell expansion and tubulin, actin, or actin-depolymerizing factors (ADF) were all highly abundant in our dataset. The GO analysis of ESTs and unigenes showed that the microtubule was the most abundant in cellular components and microtubule-based movement was the third richest in biological process (Table S17). Study results indicated that the accumulation of various cell cytoskeleton-related transcripts will undoubtedly contribute to the rapid elongation of fiber cells [17], [45], [46], [59].

Through comparative analysis of expression profiling, Chaudhary et al. [13], [62] and Hovav et al. [25] found polyploidy, human selection, and domestication-enhanced modulation of cellular redox levels and the avoidance or delay of stress-like processes, which prolonged the elongation period and growth of longer fiber. ROS were continuously produced by oxidases or by electron transport components, such as FeS centers, semiquinones, or ferredoxin. However, large pools of glutathione and ascorbate were maintained in a highly reduced state in nonquiescent cells under optimal conditions. Other key redox signaling components were thioredoxins (TRX) and glutaredoxins (GRX), which were reduced by ferredoxin, NADPH, or glutathione [63]. Given the previous evidence that cotton class III peroxidases (GhPOX1) and ascorbate peroxidase (GhAPX1) play important roles during fiber cell elongation possibly by mediating ROS homeostasis [20], [64]. The classification and assignment protein motif/domain for ESTs and unigenes showed that NAD(P)^+^, thioredoxin, ferredoxin, and glutathione-related domains were the richest protein domain (Table 3 and Table S12). There are about 580 ESTs with the GO annotation of “biology process∶oxidation reduction” (Figure S4), which was more abundant than other process. The recent study indicated that ROS also affect fiber initiation [26].

### Expression profile analysis might help find species-specific unigenes from *G. barbadense*

Through a similar search against other three cotton species genomic resources, 87.4% of unigenes have similarity against *G. hirsutum* and 2991 (51.1%) were shared by all four species; however, these sequences are more nearly similar with *G. hirsutum* (Fig. 3a). Finally, 669 (11.4%) had no match to any sequences in current EST databases of cotton. Blastx against protein datasets of *Arabidopsis* and GO annotation showed that the most enrichment functions were transport and oxidoreductase activity (Table S13 and Table S17). Moreover, several *G. barbadense-*specific genes deserve to be highlighted. The contig sequence CO000339 (similar with AT3G14130) encoded (S)-2-hydroxy-acid oxidase, peroxisomal, which is mitochondrial type II peroxiredoxin F and essential for redox homeostasis and root growth of *Arabidopsis thaliana* under stress [65]. Some genes involved in the stress response process, such as contigs CO000432 encoded pyruvate orthophosphate dikinase (PPDK), which is involved in a metabolic response to water deficit and low-oxygen stress in rice, an anoxia-tolerant species [66]. Soluble epoxidehydrolase (AtSEH) gene (AT2G26740, similar with contigs CO000715) concerned with epoxide hydrolase lipid metabolism, which showed a relatively high expression in response to malondialdehyde treatment [67]. PDX1 (protein heterodimerization, AT3G16050) was essential for vitamin B_6_ biosynthesis, development, and stress tolerance in *Arabidopsis* [68]. Two other important genes were vital for trichomes, contig CO001421 (similar with AT3G03980) encoded short-chain dehydrogenase/reductase (SDR) family protein, which is up-regulated both in *Arabidopsis* trichomes and root atrichoblasts [69]. *Arabidopsis* WAVE complex subunit (AT2G34150.2) activates the Arp2/3 complex and was required for epidermal morphogenesis [70]. In addition, another two genes were related to auxin, IBA-RESPONSE 3 (IBR3, AT3G06810, similar with contig CO001432) and IAA-ALANINE RESISTANT 3(IAR3). IBR3 may act directly in the oxidation of IBA to IAA [71]. The mutant deficiency of IBR3 failed to expand root hairs and exogenous active auxin restored its root hair elongation [72]. IAR3 encoded an auxin conjugate hydrolase [73]. Moreover, contigs CO000682 (similar with AT1G67710) encoded RESPONSE REGULATOR 11 (ARR11) TF, which belonged to the cytokinin-associated type-B ARR subfamily and had an essential role in cytokinin signal transduction [74]. These *G. barbadense-*specific sequences perhaps contribute to the differences between *G. barbadense* and other cotton species.

## Materials and Methods

### EST generation

The construction of the normalized fiber cDNA library (from −2 to 25 dpa) of *G. barbadense* cv. 3–79 (the genetic standard line) by saturation hybridization with genomic DNA was been described by Tu et al. [12]. The clones were randomly picked and transferred into 384-well plates. Single-pass sequencing from the 5′ end was carried out with ABI 3730 automatic DNA sequencer (Auke Biotech Co., Ltd., Beijing, China) by using T3 universal primer and BigDye Terminator.

### EST pre-process and assembly

The trace files were base-called using Phred program [75], [76] and all low-quality bases (<Q20, <99% accuracy) were removed from the sequence ends, followed by SeqClean [77] to shorten Poly-A/T (only hold 5 continual bases A/T). Then the vector and contaminating microbial sequences were eliminated using VecScreen program (http://www.ncbi.nlm.nih.gov/VecScreen/VecScreen.html). EST sequences longer than 100 bp after trimming were deposited into the dbESTs division of GenBank, then clustered and assembled into contigs and singlets (unisequences) using ESTClustering [78], [79], which was designed based on MegaBlast [79] and CAP3 [80].

### Annotation and functional classification

After clustering and assembly, BLAST search was done to identify similarities between the ESTs and sequences deposited in public databases. All of the unisequences were then compared to SwissProt and GenBank non-redundant protein and nucleotide databases using either blastx (E-value≤10^−5^) or blastn (E-value≤10^−5^) program [81]. The ESTs of *G. hirsutum*, *G. raimondii*, and *G. arboretum* species from dbEST at NCBI were downloaded and blastn analysis was performed to compare *G. barbadense* unisequences obtained in this study. In addition, the unigenes were compared with the protein sequences of *A. thaliana* (http://www.Arabidopsis.org/), *O. sativa* (http://rice.plantbiology.msu.edu/), *P. trichocarpa* (http://genome.jgi-psf.org/poplar/), *V. vinifera* (http://www.genoscope.cns.fr/externe/), and *R. communis* (http://castorbean.jcvi.org/) using blastx. The relative similarity relationships between *G. barbadense* with other species were displayed using the program SimiTri [34]. To identify putative cell wall-related genes and transcription factors, the blastx against Cell Wall Navigator (CWN) database [35] and an comprehensive plant transcription factor database (PlantTFDB) [36], [82] were used.

Gene Ontology (GO) [83] annotation was performed with BLAST2GO [30], [31] based on sequence similarity. For the annotation, the default configuration settings were used (blastx against NCBI non-redundant (nr) protein database, E-value filter ≤10^−3^, HSP length cutoff of 33, maximum 20 BLAST hits per sequence to sequence description tool and annotation cutoff of 55). Furthermore, to improve annotability, InterProScan was performed and InterProScan results were merged to GO annotation. Then, the GOslim “goslim_plant.obo” was used to achieve plant-related GO terms. Finally, the analysis of biological processes/pathways was also carried out using the KEGG (Kyoto Encyclopedia of Genes and Genomes) [84] Automatic Annotation Server [33] with the SBH option checked and plant gene datasets selected.

BLAST2GO includes the Gossip package [85] for statistical assessment of annotation differences between two sets of sequences, using Fisher's exact test for each GO term. FDR controlled *P* values (FDR<0.05) were used for the assessment of differentially significant metabolic pathways.

### Identification of EST-SSRs

The EST-SSRs were indentified using Serafer, a visualization powerful pipeline to assemble sequences, detect SSRs, and design primers, developed by Shaoguang Liang in our laboratory (ftp://ensembl.genomics.org.cn/other/Serafer_1.9.5.zip or http://www.tgir.org/download/software/Serafer_1.9.5.zip) (unpublished). The length criteria for SSR detection were a minimum of seven repeats for dinucleotide motifs, five repeats for trinucleotide motifs, and four repeats for tetra-, penta-, and hexanucleotide motifs.

## Supporting Information

Figure S1 **The length distribution of ESTs, contigs, singletons, and unigenes.**
(PDF)

Figure S2 **Expression analysis of 15 representative unigenes by RT-PCR method.** The first ten are selected from the list of enriched unigenes, the latter five are from the list of G. barbadense putative specific sequences. R, L, S, 0, 5, 10, 15 and 20 represents the tissue of root, leaf, stem and 0, 5, 10, 15, 20-days post anthesis (DPA) fibers.
(PDF)

Figure S3 **Functional classifications for the 5852 unigenes that were assigned with GO terms (third level GO terms).** The three GO categories, biological process (a), molecular function (b), and cellular component (c) are presented.
(PDF)

Figure S4 **The GO distribution of 10,979 ESTs.**
(PDF)

Table S1 **The length distribution of ESTs, contigs, singletons, and unigenes.**
(XLS)

Table S2 **Ninety three highly abundant genes in the 10,979 ESTs (ESTs≥10).**
(XLS)

Table S3 **The primers used in RT-PCR analysis.**
(PDF)

Table S4 **Details of best blastx hits against nr with E-value≤10^−5^.** The 5852 unique sequences were used in a blastx search against the non-redundant protein sequence (nr) database in GenBank. A total of 4862 (83.1%) unigenes had significant hits (E-value≤10^−5^).
(XLS)

Table S5 **List of unigenes without blastx result.** 990 (16.9%) of 5852 unique sequences showed no significant similarity to any sequences contained in the nr database.
(XLS)

Table S6 **Species distribution of best blastx hits.** The organism distribution of the unigenes best blastx hits for the 4862 unigenes.
(XLS)

Table S7 **Details of best blastx hits against nr and swissprot database E-value≤10^−5^ of the 2697** ***G. barbadense*** **unigenes which have similarity with unknown function proteinsof nr database in GenBank.**
(XLS)

Table S8 **Species distribution of unknown function proteins that have the best similarity with 2697** ***G. barbadense*** **unigenes.**
(XLS)

Table S9 **Details of best blastn result against nt with E-value≤10^−5^.** 990 sequences with no blastx hits were searched for similarities at the nucleotide level (non-redundant nucleotide sequence nt database in GenBank). 205 sequences shared homology with the genes registered in the NCBI nt database.
(XLS)

Table S10 **Details of best blastn result species of** ***Gossypium*** **.** 990 sequences with no blastx hits were searched for similarities at the nucleotide level. 106 sequences have similarity to cotton.
(XLS)

Table S11 **List of unigenes number and length without blastn result.** 990 sequences with no blastx hits were searched for similarities at the nucleotide level. 785 unigenes (13.4%) remained unidentified.
(XLS)

Table S12 **Detailed result of InterProScan.** The function motif/domain for ESTs and unigenes were obtained through InterProScan use of BLAT2GO bioinformatics tool.
(XLS)

Table S13 **669** ***G. barbadense-*** **specific unigenes blastx against** ***Arabidopsis*** **.** 669 unigenes without significant match to any sequence in the current EST databases of cotton, was used in a blastx search against the protein datasets of *Arabidopsis* with E-value≤10^−5^.
(XLS)

Table S14 **List and classification of cell wall-related genes.** The 5852 unigenes were assessed by blastx against the Cell Wall Navigator (CWN) database to identify cell wall-related genes. 282 sequences had homologs (blastx, E-value≤10^−5^) in the CWN.
(XLS)

Table S15 **List and categories of putative transcription factors.** The 5852 unigenes were assessed by blastx against the PlantTFDB 2.0 database to identify putative TFs. 736 sequences (12.6% of unigenes, including 1317 ESTs) had homologs in PlantTFDB at E-value≤10^−5^.
(XLS)

Table S16 **Number of dinucleotide and trinucleotide repeats.**
(XLS)

Table S17 **669** ***G. barbadense-*** **specific unigenes statistical distinct GO terms against the other 5183 unigenes.** Gene Ontology (GO) annotation was performed with BLAST2GO. Then, the Gossip package was used to get statistical assessment of annotation differences between two sets of sequences, using Fisher's exact test for each GO term. FDR controlled P values (FDR<0.05) were used.
(XLS)

File S1 **The details of expression analysis by RT-PCR method.** Plant materials, RNA extraction and RT-PCR details were described in this file.
(PDF)
