# Supplementary figures and images for: Generation, Annotation and Analysis of First Large-Scale Expressed Sequence Tags from Developing Fiber of Gossypium barbadense L
Source: PLoS One. 2011 Jul 28;6(7):e22758. doi: 10.1371/journal.pone.0022758 (PMC3145671; doi:10.1371/journal.pone.0022758)

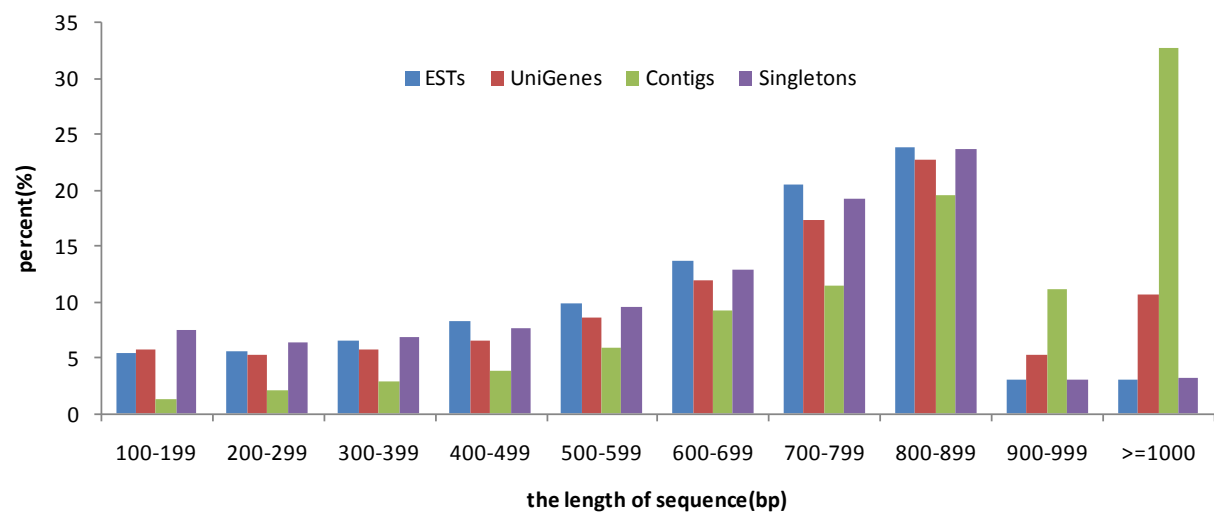

**Figure S1 the length distribution of sequences**

Supplement: Figure S1 — The length distribution of ESTs, contigs, singletons, and unigenes. (PDF) [file pone.0022758.s001.pdf]
