# Supplementary material for: Generation, Annotation and Analysis of First Large-Scale Expressed Sequence Tags from Developing Fiber of Gossypium barbadense L
Source: PLoS One. 2011 Jul 28;6(7):e22758. doi: 10.1371/journal.pone.0022758 (PMC3145671; doi:10.1371/journal.pone.0022758)
Supplement: Figure S2 — Expression analysis of 15 representative unigenes by RT-PCR method. The first ten are selected from the list of enriched unigenes, the latter five are from the list of G. barbadense putative specific sequences. R, L, S, 0, 5, 10, 15 and 20 represents the tissue of root, leaf, stem and 0, 5, 10, 15, 20-days post anthesis (DPA) fibers. (PDF) [file pone.0022758.s002.pdf]

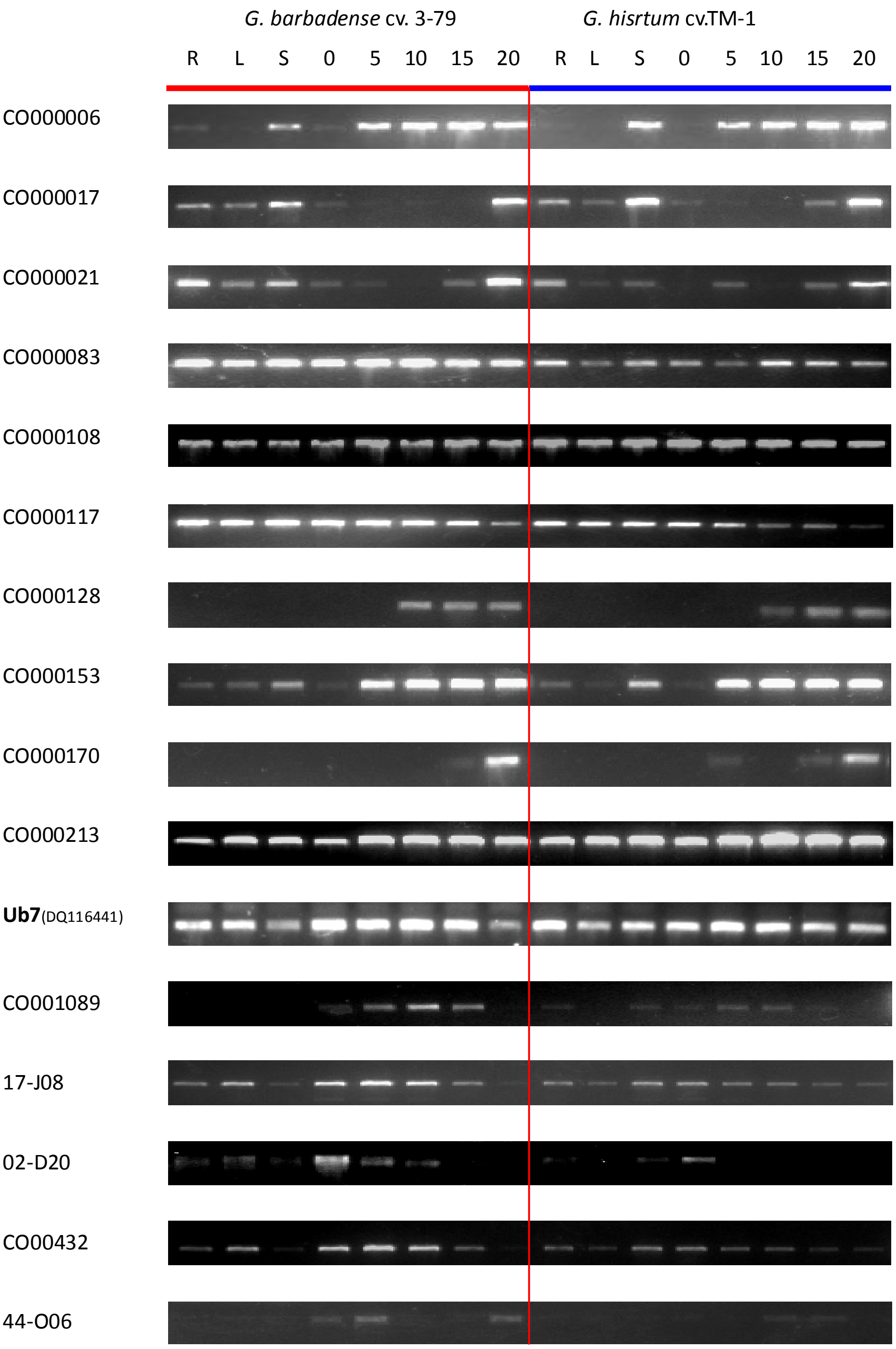

Figure S2. Expression analysis of 15 representative unigenes by RT-PCR method. The first ten are selected from the list of enriched unigenes, the latter five are from the list of G. barbadense putative specific sequences. R, L, S, 0, 5, 10, 15 and 20 represents the tissue of root, leaf, stem and 0, 5, 10, 15, 20-days post anthesis (DPA) fibers.
