# Supplementary material for: Generation, Annotation and Analysis of First Large-Scale Expressed Sequence Tags from Developing Fiber of Gossypium barbadense L
Source: PLoS One. 2011 Jul 28;6(7):e22758. doi: 10.1371/journal.pone.0022758 (PMC3145671; doi:10.1371/journal.pone.0022758)
Supplement: Figure S3 — Functional classifications for the 5852 unigenes that were assigned with GO terms (third level GO terms). The three GO categories, biological process (a), molecular function (b), and cellular component (c) are presented. (PDF) [file pone.0022758.s003.pdf]

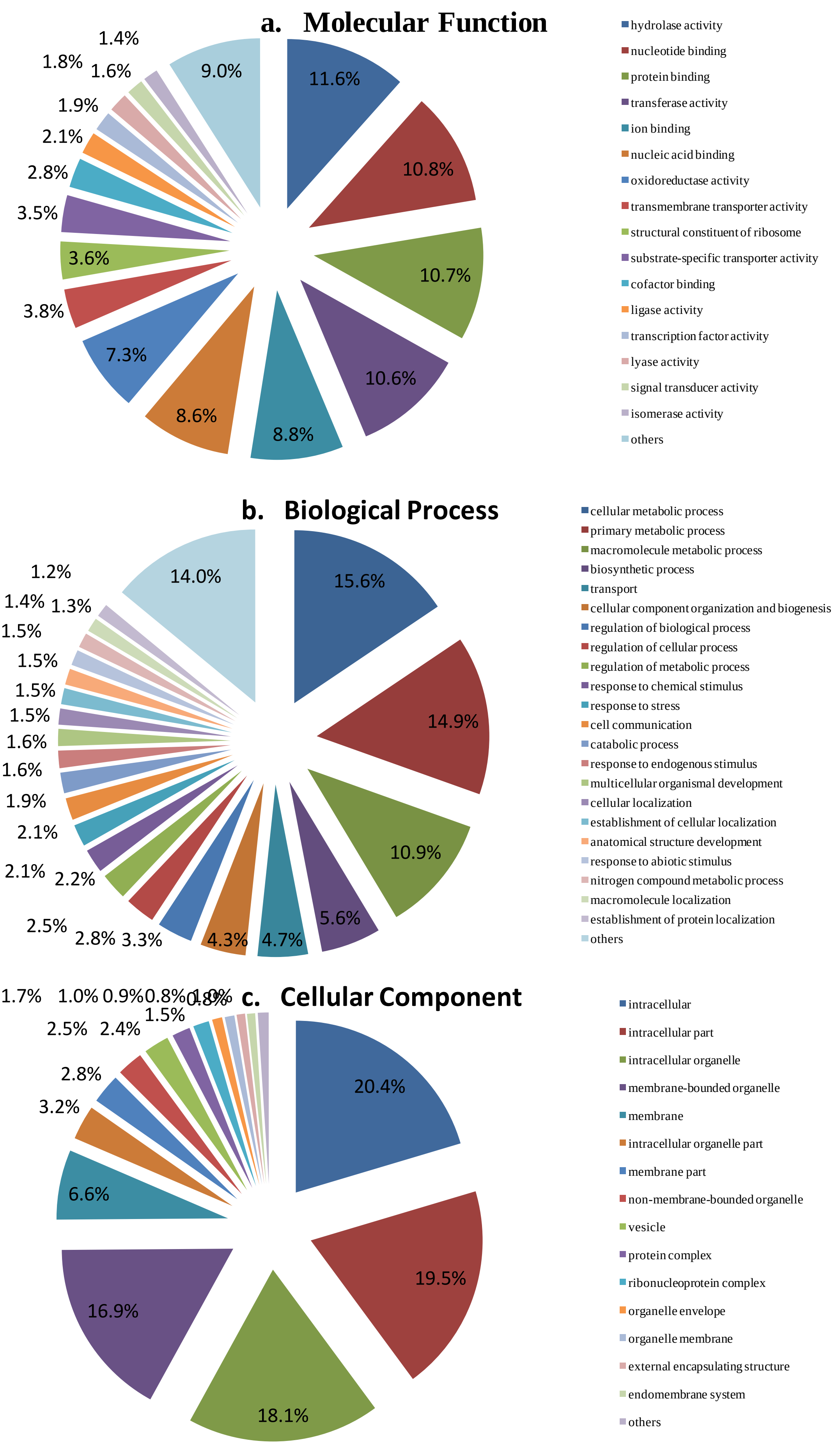

Figure S3 Functional classifications for the 5,852 unigenes which were assigned with GO terms (third level GO terms). The three GO categories, biological process (a), molecular function (b) and cellular component (c) are presented.
