# Supplementary material for: Generation, Annotation and Analysis of First Large-Scale Expressed Sequence Tags from Developing Fiber of Gossypium barbadense L
Source: PLoS One. 2011 Jul 28;6(7):e22758. doi: 10.1371/journal.pone.0022758 (PMC3145671; doi:10.1371/journal.pone.0022758)
Supplement: Figure S4 — The GO distribution of 10,979 ESTs. (PDF) [file pone.0022758.s004.pdf]

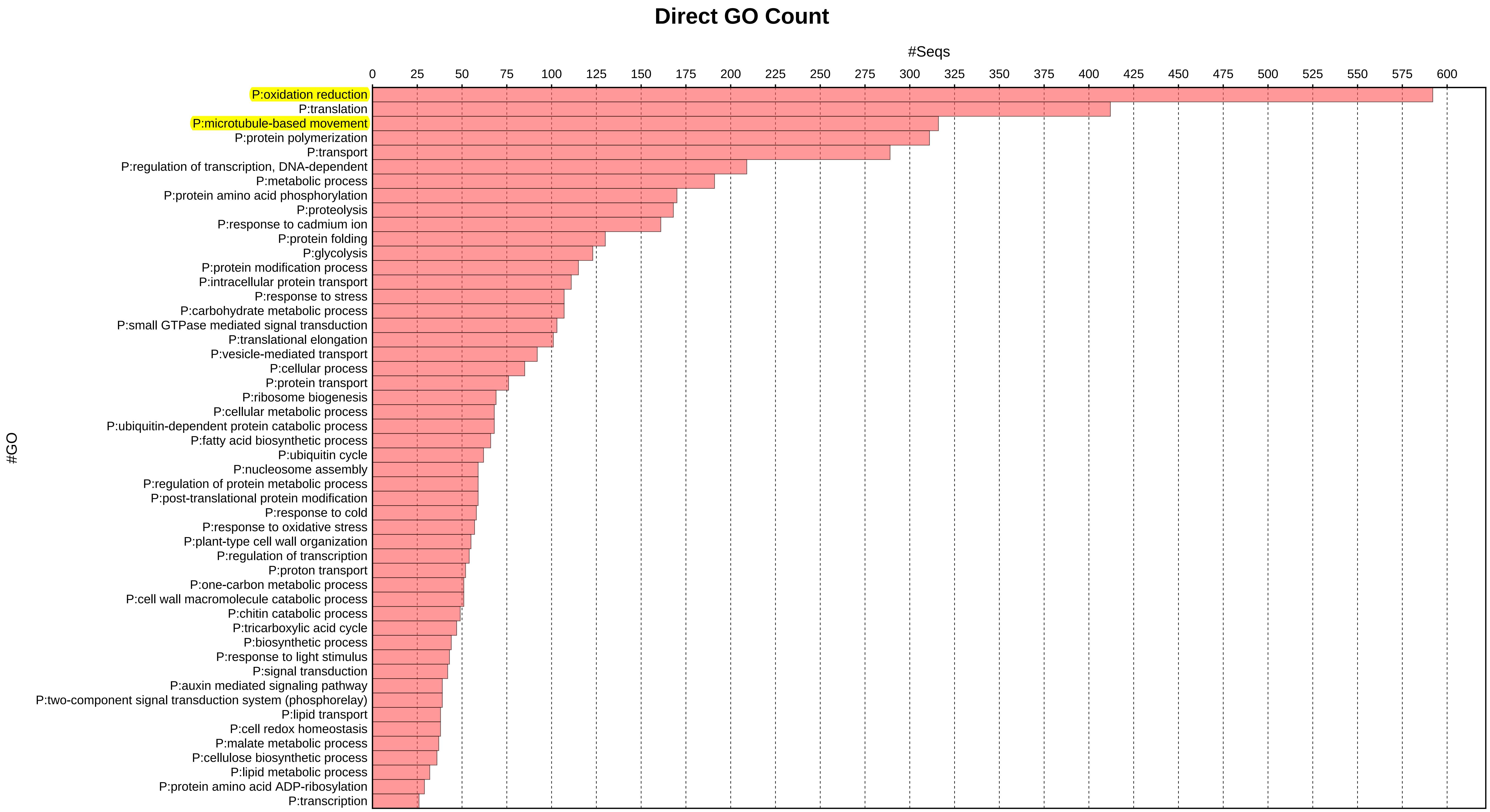
Direct GO Count
#Seqs
0
25
50
75
100
125
150
175
200
225
250
275
300
325
350
375
400
425
450
475
500
525
550
575
600
#GO
P:oxidation reduction
P:translation
P:microtubule-based movement
P:protein polymerization
P:transport
P:regulation of transcription, DNA-dependent
P:metabolic process
P:protein amino acid phosphorylation
P:proteolysis
P:response to cadmium ion
P:protein folding
P:glycolysis
P:protein modification process
P:intracellular protein transport
P:response to stress
P:carbohydrate metabolic process
P:small GTPase mediated signal transduction
P:translational elongation
P:vesicle-mediated transport
P:cellular process
P:protein transport
P:ribosome biogenesis
P:cellular metabolic process
P:ubiquitin-dependent protein catabolic process
P:fatty acid biosynthetic process
P:ubiquitin cycle
P:nucleosome assembly
P:regulation of protein metabolic process
P:post-translational protein modification
P:response to cold
P:response to oxidative stress
P:plant-type cell wall organization
P:regulation of transcription
P:proton transport
P:one-carbon metabolic process
P:cell wall macromolecule catabolic process
P:chitin catabolic process
P:tricarboxylic acid cycle
P:biosynthetic process
P:response to light stimulus
P:signal transduction
P:auxin mediated signaling pathway
P:two-component signal transduction system (phosphorelay)
P:lipid transport
P:cell redox homeostasis
P:malate metabolic process
P:cellulose biosynthetic process
P:lipid metabolic process
P:protein amino acid ADP-ribosylation
P:transcription

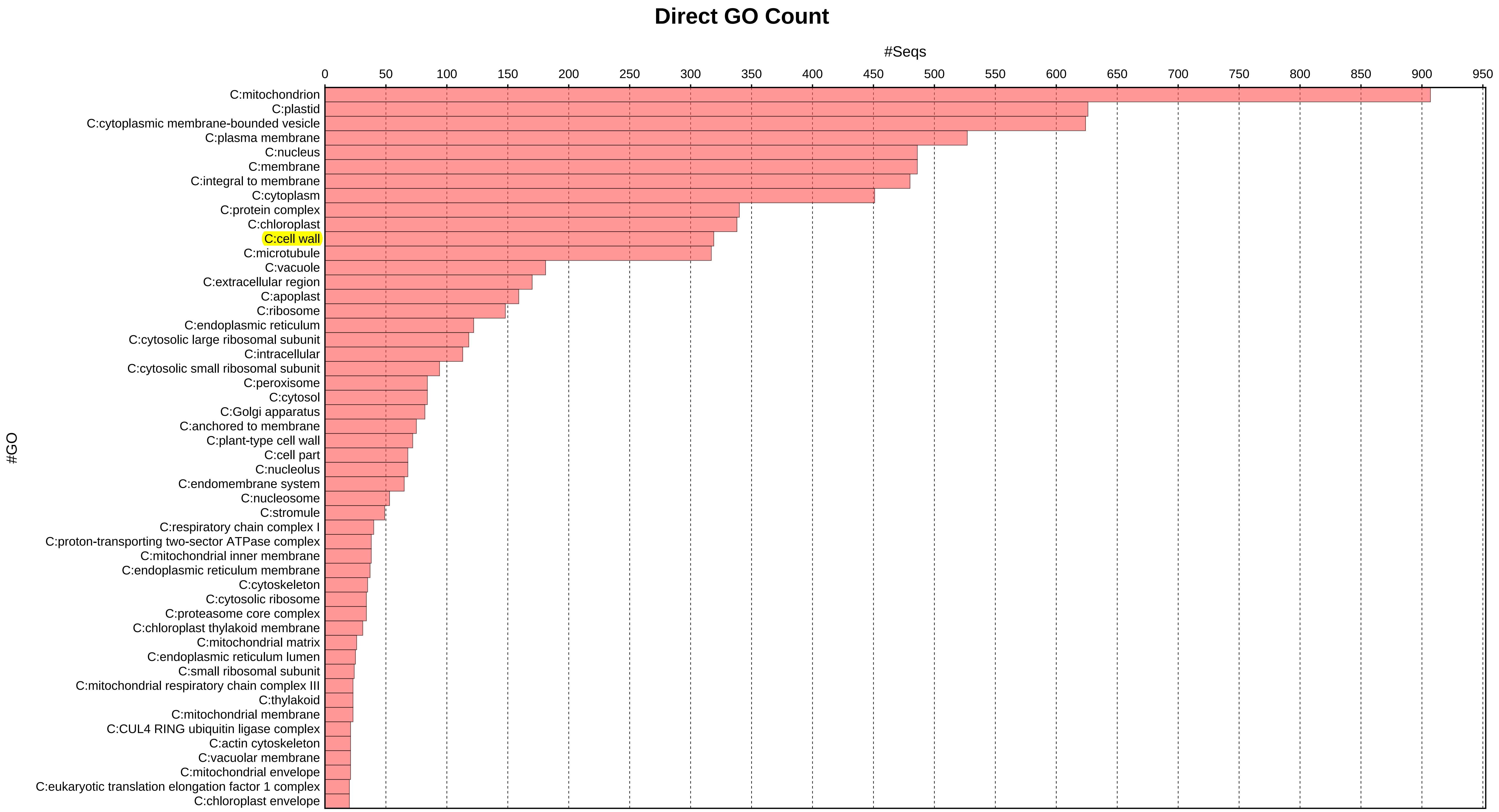

Direct GO Count
#Seqs
0
50
100
150
200
250
300
350
400
450
500
550
600
650
700
750
800
850
900
950
#GO
C:mitochondrion
C:plastid
C:cytoplasmic membrane-bounded vesicle
C:plasma membrane
C:nucleus
C:membrane
C:integral to membrane
C:cytoplasm
C:protein complex
C:chloroplast
C:cell wall
C:microtubule
C:vacuole
C:extracellular region
C:apoplast
C:ribosome
C:endoplasmic reticulum
C:cytosolic large ribosomal subunit
C:intracellular
C:cytosolic small ribosomal subunit
C:peroxisome
C:cytosol
C:Golgi apparatus
C:anchored to membrane
C:plant-type cell wall
C:cell part
C:nucleolus
C:endomembrane system
C:nucleosome
C:stromule
C:respiratory chain complex I
C:proton-transporting two-sector ATPase complex
C:mitochondrial inner membrane
C:endoplasmic reticulum membrane
C:cytoskeleton
C:cytosolic ribosome
C:proteasome core complex
C:chloroplast thylakoid membrane
C:mitochondrial matrix
C:endoplasmic reticulum lumen
C:small ribosomal subunit
C:mitochondrial respiratory chain complex III
C:thylakoid
C:mitochondrial membrane
C:CUL4 RING ubiquitin ligase complex
C:actin cytoskeleton
C:vacuolar membrane
C:mitochondrial envelope
C:eukaryotic translation elongation factor 1 complex
C:chloroplast envelope

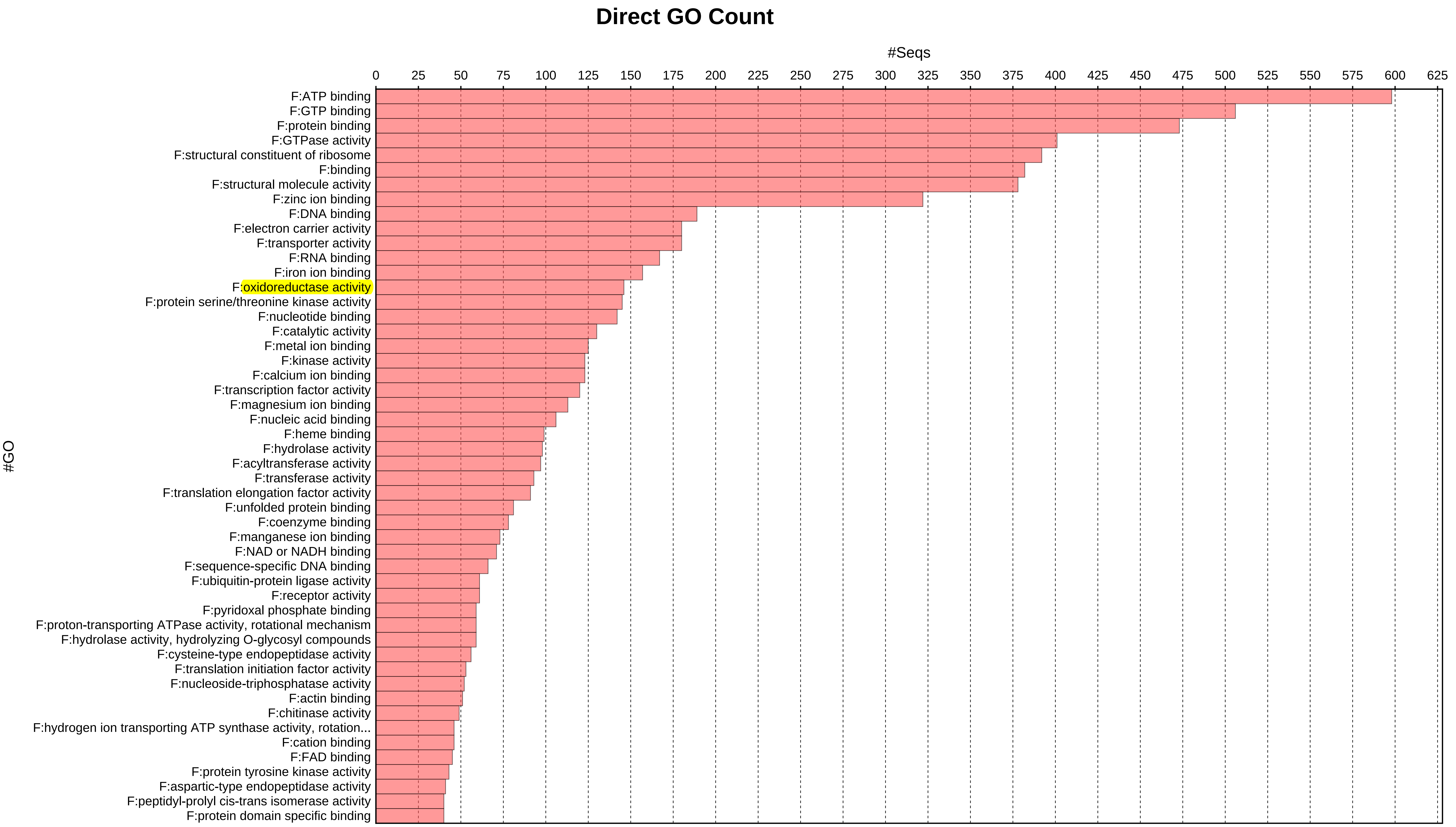

Direct GO Count
#Seqs
0
25
50
75
100
125
150
175
200
225
250
275
300
325
350
375
400
425
450
475
500
525
550
575
600
625
#GO
F:ATP binding
F:GTP binding
F:protein binding
F:GTPase activity
F:structural constituent of ribosome
F:binding
F:structural molecule activity
F:zinc ion binding
F:DNA binding
F:electron carrier activity
F:transporter activity
F:RNA binding
F:iron ion binding
F:oxidoreductase activity
F:protein serine/threonine kinase activity
F:nucleotide binding
F:catalytic activity
F:metal ion binding
F:kinase activity
F:calcium ion binding
F:transcription factor activity
F:magnesium ion binding
F:nucleic acid binding
F:heme binding
F:hydrolase activity
F:acyltransferase activity
F:transferase activity
F:translation elongation factor activity
F:unfolded protein binding
F:coenzyme binding
F:manganese ion binding
F:NAD or NADH binding
F:sequence-specific DNA binding
F:ubiquitin-protein ligase activity
F:receptor activity
F:pyridoxal phosphate binding
F:proton-transporting ATPase activity, rotational mechanism
F:hydrolase activity, hydrolyzing O-glycosyl compounds
F:cysteine-type endopeptidase activity
F:translation initiation factor activity
F:nucleoside-triphosphatase activity
F:actin binding
F:chitinase activity
F:hydrogen ion transporting ATP synthase activity, rotation...
F:cation binding
F:FAD binding
F:protein tyrosine kinase activity
F:aspartic-type endopeptidase activity
F:peptidyl-prolyl cis-trans isomerase activity
F:protein domain specific binding
