# Supplementary material for: Generation, Annotation and Analysis of First Large-Scale Expressed Sequence Tags from Developing Fiber of Gossypium barbadense L
Source: PLoS One. 2011 Jul 28;6(7):e22758. doi: 10.1371/journal.pone.0022758 (PMC3145671; doi:10.1371/journal.pone.0022758)
Supplement: Table S3 — The primers used in RT-PCR analysis. (PDF) [file pone.0022758.s007.pdf]

Table S3. The primers used in RT-PCR analysis

Sequence Number	Forward primer (5′-3′)	Reverse primer(5′-3′)
CO000006	TCTCGTCTCTGGGTCTCGTAAGT	GGCTACTCCCCTCATCACCAG
CO000017	TGGGTTCTGGGATTACCATTCTT	ACCTTCAATGCGTCGCCTGT
CO000021	CTTTTGGGTATTGCTTGGGCT	CGCAAGTGCTCCCACCATT
CO000083	ACATAACTGTCAACGAAGCCCAAT	CAAGCCCACTTTGAAACCCTAA
CO000108	GCACCTGGACACCGTGACTTTAT	TTTCAACACGACCAACAGGGAC
CO000117	AAGGCTCCTCTTTCCATCGTGT	ACCACCAAACCCAGTGACGATA
CO000128	TCAGGGAATGGTGATGGTGC	CAATGTAGCAAGCAAGCCAATC
CO000153	ACTCCTCTTCCCACTTCATCACC	ATTTGCTCCGCTCGTGTCTGT
CO000170	GCTACGGTGCCTGTTTATGGG	CACAATGGAACCTTCTGGGCTTA
CO000213	GTGGCTACCCTTCCAACTGTGA	TTCTTGAACCACATTTAGCCCTG
**Ub7(DQ116441)**	GAAGGCATTCCACCTGACCAAC	CTTGACCTTCTTCTTCTTGTGCTTG
CO001089	CTGTTCTTTCAGCCTGTGCCG	GCTTGTTCCCATTTGTTCGTATTG
17-J08	CAAAAATCGAAAATGCCTCTGG	AGCTACCTGAAGCTCGCTTAAAG
02-D20	CAAAGATCCTACATCTGTGCCTCC	ACTTGGGAACTGACTACTATCTGCC
CO00432	GCCGTAGAAGTGTTCGCCAAT	CAACTCGCCGCAAATGCC
44-O06	AAGCGGAAGGTCTCGGAAAT	AAAGAGGCTCAGGCAAGTTCG
