# Supplementary material for: Generation, Annotation and Analysis of First Large-Scale Expressed Sequence Tags from Developing Fiber of Gossypium barbadense L
Source: PLoS One. 2011 Jul 28;6(7):e22758. doi: 10.1371/journal.pone.0022758 (PMC3145671; doi:10.1371/journal.pone.0022758)
Supplement: File S1 — The details of expression analysis by RT-PCR method. Plant materials, RNA extraction and RT-PCR details were described in this file. (PDF) [file pone.0022758.s022.pdf]

Plant materials

Gossypium barbadense cv. 3-79 (the genetic standard line) and *G. hirsutum* genetic standard line Texas Marker-1 (TM-1) were cultivated in the field with normal farming practices. The bolls were tagged on the day of anthesis. Fiber tissues were obtained 0, 5, 10, 15 and 20-days post anthesis (DPA) from field grown plants. Fibers were isolated carefully from ovules and immediately submerged in liquid nitrogen. Root, leaf and stem tissues were harvested from two true leaves old plants grown in a growth chamber (16 h light/8 h dark, 35℃/26℃). All selected materials were stored at -70℃ after being frozen in liquid nitrogen until use.

RNA extraction and RT-PCR

RNA was extracted using a modified guanidine thiocyanate method (Zhu et al. 2005). These RNA samples (3 μg) were reverse-transcribed to cDNA by using the Superscript III RT (Invitrogen, San Diego, USA). Products of each biosynthesis were diluted to 1 000 μl before the PCR procedure. Primers for the RT-PCR were designed using Primer Premier 5.0. RT-PCR was performed in 20 μl reactions using 5 μl first-strand cDNA as template. As a control, the UBQ7 (gi number: DQ116441) was used as the endogenous reference gene (Tu et al. 2007). The PCR program was denaturized at 94℃ for 3 min, followed by 28 cycles of 94℃ for 30 s, 58℃ for 30 s, and 72℃ for 30 s, and then a final extension at 72℃ for 5 min. One to twelve additional cycles were added for some lower expression genes. The PCR products (8 μl) for each sample were then electrophoresed in a 2% ethidium bromide agarose gel and viewed under ultraviolet light.

Tu L, Zhang X, Liu D, et al. (2007). Suitable internal control genes for qRT-PCR normalization in cotton fiber development and somatic embryogenesis. Chinese Science Bulletin 52(22): 3110-3117

Zhu L F, Tu L L, Zeng F C, et al. (2005). An improved simple protocol for isolation of high quality RNA from Gossypium spp. suitable for cDNA library construction. Acta Agronomica Sinica, 31,1657-1659
